# Patterns of urinary albumin and IgM associate with markers of vascular ageing in young to middle-aged individuals in the Malmö offspring study

**DOI:** 10.1186/s12872-020-01638-3

**Published:** 2020-08-05

**Authors:** Per Swärd, Rafid Tofik, Omran Bakoush, Ole Torffvit, Peter M. Nilsson, Anders Christensson

**Affiliations:** 1 Department of Orthopedics, Clinical Sciences, Clinical and Molecular Osteoporosis Research Unit, Lund University, Skåne University Hospital, 205 02 Malmö, SE Sweden; 2 Department of Emergency medicine, Clinical Sciences, Lund University, Skåne University Hospital, Malmö, Sweden; 3 Department of Nephrology, Clinical Sciences, Lund University, Skåne University Hospital, Lund, Sweden; 4 Department of Internal Medicine, College of Medicine and Health sciences, UAEU, Al Ain, United Arab Emirates; 5 Department of Nephrology, Clinical Sciences, Lund University, Skåne University Hospital, Lund, Sweden; 6 Department of Clinical Sciences and Internal Medicine, Lund University Skane University Hospital, Malmo, Sweden; 7 Department of Nephrology, Clinical Sciences, Lund University, Skåne University Hospital, Malmö, Sweden

**Keywords:** Ankle-brachial-index, Arteriosclerosis, Albumin creatinine ratio, Atherosclerosis, Glomerular filtration barrier, Pulse wave velocity, Microalbuminuria, Urinary IgM

## Abstract

**Background:**

Increased urinary excretion of IgM and low-grade albuminuria are associated with increased risk of cardiovascular morbidity and mortality. The objective of this study was to investigate the association between urinary IgM, albuminuria, and vascular parameters reflecting arterial structure and function.

**Methods:**

Subjects of the present study were from the Malmö Offspring study (MOS) cohort, and included 1531 offspring (children and grand-children) to first-generation subjects that participated in the Malmö Diet Cancer-Cardiovascular Arm study cohort. At baseline, technical measurements of arterial stiffness (carotid-femoral pulse wave velocity; c-f PWV), carotid arterial morphology, 24-h ambulatory blood pressure recordings, ankle-brachial-index (ABI), and evaluation of endothelial function (reactive hyperemia index, RHI) were performed. Urinary (U) IgM, U-albumin, and U-creatinine were measured. Multivariate adjusted logistic regression was used to test whether U-IgM excretion and increasing urinary albumin excretion were related to vascular parameters.

**Results:**

Detectable U-IgM was independently associated with higher systolic blood pressure, odds ratio (OR) 1.021, 95% confidence interval (CI, 1.003–1.039), *p* = 0.025 and lower ABI; ABI dx: OR 0.026, 95% CI (0.002–0.381), *p* = 0.008, ABI sin: OR 0.040, 95% CI (0.003–0.496), *p* = 0.012. Low-grade albuminuria was independently associated with systolic and diastolic blood pressure, aortic blood pressure, the c-f PWV and the number of carotid intima plaques (*p* < 0.05).

**Conclusions:**

In young to middle-aged, mostly healthy individuals, increased U-IgM excretion and low-grade albuminuria are associated with adverse vascular parameters. Increased U-IgM excretion may reflect subclinical peripheral atherosclerosis, whereas increased U-albumin excretion is associated with a wide range of cardiovascular abnormalities. This may reflect different pathophysiological mechanisms.

## Background

Several biomarkers have been shown to be prognostic of cardiovascular risk, but most do not improve clinical prediction when combined with traditional risk factors; age, sex, hypertension, diabetes, blood lipids and smoking. Some of the most prominent markers of cardiovascular risk which have also been shown to independently predict cardiovascular events, include impaired kidney function [1, 2], large artery stiffening [3–5], the ankle-brachial index, ABI (a marker of peripheral atherosclerosis) [6–8], as well as markers of injury to the glomerular filtration barrier (GFB) [9–11].

Low grade albuminuria/microalbuminuria (3–30 mg albumin/mmol creatinine or 30–300 mg albumin/g creatinine), or 20 to 200 μg albumin excreted in the urine per minute (daytime)) is well-known to predict cardiovascular events and mortality in various clinical cohorts, but also in subjects with hypertension and diabetes from the general population [12]. These findings are independent of renal function (estimated glomerular filtration rate; eGFR), and are believed to represent a link between albuminuria, atherosclerosis and systemic endothelial dysfunction [11, 13, 14].

In middle-aged and elderly individuals, levels of urinary albumin excretion > 6.8 μg albumin excreted in the urine per minute (daytime) was associated with cardiovascular morbidity and mortality in the general population [13]. Abnormal atherosclerotic conditions, i.e. arterial stiffness, carotid plaques and peripheral artery disease, was more common at a U-albumin/creatinine ratio (U-ACR) > 15 mg/g (~ equivalent to 1,5 mg albumin/mmol creatinine) [15]. However, the association between normoalbuminuria/ microalbuminuria and abnormal cardiovascular conditions such as arterial stiffness, manifest atherosclerosis and markers of endothelial dysfunction in younger populations is not well documented.

We have previously shown in clinical studies that increased urinary excretion of IgM is independently associated with increased risk of subsequent cardiovascular events and mortality in subjects with diabetes (type 1 and 2) [10, 16]. The same was true in patients with acute coronary syndrome (ACS) and in subjects presenting to the emergency room with acute chest pain, without ACS [9, 17]. Interestingly, the highest risk of cardiovascular events or mortality was observed in subjects who had a combination of increased urinary IgM excretion and low grade albuminuria [9, 10, 16]. Normally, because of its size, IgM (120 Å) does not pass the GFB, unless there are large defects in the GFB (“shunts”) [18–20]. The prevalence of increased urinary IgM excretion in the general population is unknown. Nor has it been investigated whether urinary IgM excretion is associated with abnormal cardiovascular conditions reflecting vascular ageing [21], such as arterial stiffness, manifest atherosclerosis or markers of endothelial dysfunction.

Therefore, the *aim* of the present observational study was to investigate the association between albuminuria and urinary IgM with adverse vascular parameters, including blood pressure, carotid intima plaques, carotid-femoral pulse wave velocity (c-f PWV), ankle-brachial index (ABI), and reactive hyperemia index (RHI) in an urban population. Furthermore, we assessed kidney function which could affect the association between albuminuria, urinary IgM and vascular parameters.

## Methods

Included in the present study were 1531 subjects from the Malmö Offspring study (MOS), children and grand-children, i.e. offspring, to first-generation subjects that participated in the Malmö Diet Cancer-Cardiovascular Arm (MDC-CV) study cohort at baseline [22], after identification based on official register information. Subjects were invited to a clinical and technical examination at the Clinical Research Unit (CRU), Skåne University Hospital, Malmö, during 2013 to 2017. The following baseline investigations were obtained in both children and grand-children (generations 2 and 3); age (y), height (m), weight (kg), (cm) and BMI (kg/m^2^), waist circumference (cm), blood pressure (mmHg), ABI and questionnaire data. The participants’ height (m) was measured to the nearest centimeter with the participant looking straight ahead, the legs together, and wearing indoor clothing without shoes or hats. Weight (kg) was measured using a calibrated balance beam or digital scale. Waist circumference (cm) was measured midway between the lowest rib margin and iliac crest. Blood pressure (mmHg) was measured after 10 min of rest lying down and the ABI was calculated. At the study start subjects answered detailed questions on family, medical, social and educational history by a web-based questionnaire. Information regarding smoking habits, use of antidiabetic medications, and antihypertensive treatment, was obtained from the questionnaire [23, 24]. Anti-hypertensive treatment included, thiazides, Beta blockers, renin-angiotensin-aldosterone system (RAAS) blockers and calcium antagonists, and in the study sample, *n* = 70 (4.6%) were treated with one or several anti-hypertensive drugs. Smokers were categorized as: nonsmokers, current smokers or previous smokers. In Table 1, we present percentages based on the whole cohort. Subjects who did not answer the questionnaire were considered to have responded negatively, adopting a conservative approach. U-albumin/creatinine ratio (U-ACR) was measured in 1530 (99.9%), and U-IgM was measured in 1339 (87.5%) of these subjects.

**Table 1:** Characteristics of study participants from the Malmö Offspring Study, with means (95% CI), SD, median (IQR) and proportions

	All subjects	Urinary IgM excretion		Urinary albumin excretion			
	*n* = 1531	Not detectable *n* = 1263	Urinary IgM ≥ 10 ng/L *n* = 76	Not detectable *n* = 838	U-ACR ≥ 3.0 mg/mmol *n* = 27	U-ACR 1.5–2.9 mg/mmol *n* = 40	U-ACR 0.1–1.4 mg/mmol *n* = 624
Age, years (95% CI)	39.7 (39.1–40.4), SD 13.4	39.2 (38.5–40.0), SD 13.2	40.3 (37.1–43.4), SD 13.9	41.5 (40.6–42.4), SD 13.1	46.2 (40.6–51.8), SD 13.4	40.3 (36.0–44.6), SD 13.4	37.1 (36.0–38.2), SD 13.3
Women, n (%)	791 (52)	649 (51)	45 (59)	457 (55)	13 (48)	25 (63)	295 (47)
Body mass index, kg/m^2^ (95% CI)	25.8 (25.6–26.1), SD 4.7	25.8 (25.5–26.0), SD 4.7	25.1 (24.1–26.2) SD 4.5	25.9 (25.6–25.2), SD 4.4	27.0 (24.9–29.1), SD 5.3	26.1 (24.4–27.7), SD 5.3	25.7 (25.3–26.1), SD 5.1
Smoking (current), n (%)	197 (13)	168 (13)	8 (11)	90 (11)	5 (19)	7 (18)	95 (15)
Smoking, current or previous, n (%)	527 (34)	442 (35)	24 (32)	285 (34)	14 (52)	14 (35)	213 (34)
Waist circumference, cm (95% CI)	89.5 (88.9–90.2), SD 13.8	89.5 (88.8–90.3), SD 13.9	87.8 (85.0–90.6), SD 12.2	89.5 (88.6–90.3), SD 13.1	96.0 (89.7–102.3), SD 16.2	90.1 (85.0–95.3), SD 16.2	89.4 (88.2–90.5), SD 14.4
Systolic blood pressure, mmHg (95% CI)	117.1 (116.4–117.8), SD 14.4	116.9 (116.1–117.7), SD 14.1	119.8 (116.1–123.5), SD 16.0	115.7 (114.8–116.6), SD 13.2	129.9 (122.0–137.7), SD 21.5	122.3 (115.4–129.3), SD 21.5	118.1 (116.9–119.2), SD 14.8
Diastolic blood pressure, mmHg (95% CI)	71.4 (70.9–71.8), SD 9.5	71.2 (70.7–71.8), SD 9.5	73.3 (70.8–75.8), SD 9.5	70.8 (70.2–71.4), SD 8.9	79.8 (75.1–84.4), SD 11.5	75.0 (71.0–79.1), SD 12.6	71.5 (70.7–72.3), SD 9.8
HbA_1c_, mmol/mol (IQR)	34 (IQR 31–36)	34 (IQR 31.5–37)	34 (IQR 31.5–39)	34 (IQR 32–37)	35 (IQR 31–39.5)	35 (IQR 31.5–39.5)	33 (IQR 31–36)
Total cholesterol, mmol/L (95% CI)	5.0 (4.9–5.0), SD 1.1	5.0 (4.9–5.0), SD 1.1	4.9 (4.7–5.2), SD 1.0	5.1 (5.0–5.1), SD 1.1	5.2 (4.8–5.6), SD 1.0	5.1 (4.8–5.4), SD 1.0	4.8 (4.7–4.9), SD 1.1
HDL cholesterol, mmol/L (95% CI)	1.62 (1.59–1.64), SD 0.48	1.62 (1.59–1.64), SD 0.48	1.64 (1.53–1.75), SD 0.49	1.66 (1.63–1.69), SD 0.48	1.61 (1.40–1.82), SD 0.54	1.60 (1.44–1.76), SD 0.51	1.57 (1.53–1.60), SD 0.48
LDL cholesterol, mmol/L (95% CI)	3.13 (3.08–3.17), SD 0.96	3.10 (3.05–3.16), SD 0.97	3.09 (2.89–3.28), SD 0.83	3.19 (3.12–3.25), SD 0.97	3.27 (2.95–3.60), SD 0.83	3.29 (2.95–3.62), SD 1.04	3.03 (2.95–3.10), SD 0.94
LDL/HDL (95% CI)	2.14 (2.09–2.19), SD 1.00	2.13 (2.07–2.18), SD 1.01	2.07 (1.86–2.28), SD 0.90	2.11 (2.05–2.18), SD 0.96	2.23 (1.92–2.53), SD 0.77	2.40 (1.95–2.86), SD 1.41	2.15 (2.06–2.23), SD 1.04
Triglycerides, mmol/L (95% CI)	0.9 (IQR 0.7–1.4)	0.9 (IQR 0.7–1.3)	1.0 (IQR 0.7–1.2)	0.9 (IQR 0.7–1.3)	1.2 (IQR 0.9–1.5)	1.0 (IQR 0.8–1.6)	0.9 (IQR 0.7–1.4)
Creatinine, μmol/L (95% CI)	79.5 (78.8–80.2), SD 14.1	79.5 (78.8–80.3) SD 14.2	78.4 (75.3–81.4) SD 13.2	79.7 (78.7–80.6), SD 14.1	79.3 (72.7–85.9), SD 16.7	73.7 (69.5–78.1, SD 13.9	79.6 (78.5–80.7), SD 13.9
eGFR, mL/min/ 1.73 m^2^ (95% CI)	85.3 (84.9–85.7), SD 7.9	85.6 (85.2–86.1), SD 7.9	85.2 (83.3–87.0), SD 7.9	84.7 (84.1–85.3), SD 8.5	80.3 (75.7–84.9), SD 11.6	85.8 (83.8–87.7), SD 6.0	86.4 (85.8–86.9), SD 6.8
Hypertension, n (%)	230 (15)	182 (14)	11 (14)	107 (13)	10 (37)	10 (25)	103 (17)
Diabetes, n (%)	38 (2.4)	30 (2.3)	1 (1.3)	15 (1.7)	1 (3.4)	2 (5)	20 (3.2)
Lipid lowering medication, n (%)	31 (2.0)	24 (1.9)	1 (1.3)	19 (2.2)	1 (3.4)	0 (0)	11 (1.7)
Anti-hypertensive treatment, n (%)	70 (4.6)	51 (4.0)	4 (5.2)	36 (4.3)	4 (14.8)	3 (7.5)	27 (4.3)
- Thiazides, n (%)	8 (0.5)	5 (0.3)	1 (1.3)	4 (0.4)	0 (0)	0 (0)	4 (0.6%)
- Beta blockers, n (%)	28 (1.8)	23 (1.8)	2 (2.6)	14 (1.7)	1 (3.4)	1 (2.5)	12 (1.9)
- RAAS- Blockers, n (%)	39 (2.5)	27 (2.1)	0 (0)	21 (2.5)	2 (6.9)	1 (2.5)	13 (2.1)
- Calcium antagonists (%)	20 (1.3)	15 (1.1)	1 (1.3)	9 (1.1)	1 (3.4)	1 (2.5)	9 (1.4)
History of AMI (%)	12 (0.8)	10 (0.8)	0 (0)	5 (0.6)	0 (0)	0 (0)	7 (1.1)
History of stroke (%)	12 (0.8)	7 (0.6)	1 (1.3)	3 (0.4)	2 (6.8)	0 (0)	7 (1.1)

*AMI* acute myocardial infarction, *CI* confidence interval, *eGFR* estimated glomerular filtration rate, *IQR* Interquartile range, *RAAS* renin-angiotensin-aldosterone system, *U-ACR* urinary albumin creatinine ratio

The ABI was measured using a sphygmomanometer and Doppler device (Hadeco Bidop ES-100 V3) [25]. The systolic blood pressure was measured first in the right arm, then in the right ankle, the left ankle and in the left arm. In the ankle, the pressure was measured in both *arteria dorsalis pedis* and in *arteria tibialis posterior*, and the left and right ABI was calculated by dividing the highest blood pressure in left and right ankle with the blood pressure from the arm with the highest value. Ambulatory blood pressure (24 h) and technical measurements of arterial stiffness were performed using TensioMed Arteriograph 24 (TensioMed™ Ltd., Hungary). Blood pressure was measured every 15 min during daytime (06.00–22.00) and every 30 min during night (22.00–06.00). Brachial systolic and diastolic blood pressure, aortic systolic and diastolic blood pressure, aortic c-f PWV (indirectly) and heart rate were recorded at each measurement. Subjects were excluded from analyses if more than 30% of the blood pressure recordings were missing [26, 27]. Aortic c-f PWV was measured only in children (generation 2) using applanation tonometry (SphygmoCor; AtCor Medical, West Ryde, New South Wales, Australia) as described previously [28]. 24-h blood pressure measurements were made in a random sample of the cohort. Measurements of carotid artery morphology were also performed only in children (generation 2) and performed as described previously using ultrasound examinations with Acuson Sequoia 512 (Acuson, Mountain View, CA, USA). A 7 MHz linear array transducer was used, and images of all plaques within the left right carotid artery were obtained, using fixed machine settings by experienced sonographers. A plaque was defined as a focal thickening of intima–media greater than 1.2 mm when measured from the media–adventitia interface to the intima–lumen interface [29], and subjects were considered to have a maximum of three plaques (common carotid artery, internal carotid artery and the carotid bifurcation). Evaluation of endothelial function by calculation of the reactive hyperemia index (RHI, EndoPAT; Itamar Medical Ltd., Cesarea, Israel) in a finger were made in a random sample of all included subjects, as described previously [30].

### Laboratory methods

Blood samples and urine were collected after an overnight fast. Plasma- creatinine levels were determined by an enzymatic colorimetric assay using a calibrator traceable to primary reference material with values assigned by isotope dilution mass spectrometry (IDMS) [31]. Cystatin C levels were determined by a particle-enhanced immunoturbidimetric method using a reference material traceable to the international cystatin C calibrator [32, 33]. Standard procedures were used to measure HbA_1c_, total cholesterol and HDL cholesterol at the Department of Clinical Chemistry, Skåne University Hospital, Malmö, Sweden. The Friedwald’s formula was used to calculate LDL cholesterol [34]. Estimated glomerular filtration rate (eGFR) was calculated using both creatinine and cystatin C and a mean of these two was created [32, 35]. Urine creatinine and albumin were measured on fresh urine samples taken at study start using standard procedures at the Department of Clinical Chemistry, Skåne University Hospital. Urine albumin concentration was measured by immunoturbidometry and the urine albumin/creatinine ratio (ACR) was calculated. All urine samples were stored at − 80 °C at the Region Skåne Biobank (BD47) before measurement of urine IgM, and the urine IgM/creatinine ratio was calculated. Urinary albumin excretion was defined as microalbuminuria (ACR > 2.9 mg/mmol) and high or low normoalbuminuria, using a cut-off at ACR 1.5 mg/mmol, based on findings from Lee et al. [15]. U-IgM was measured with an in-house ELISA method, described in detail elsewhere [36]*.* The lower detection limit for the U-IgM assay is 10 ng/l. The proportion of subjects that have undergone the various examinations performed in the present study is shown in Supplementary Table 1.

### Statistical methods

Data is presented as mean and 95% confidence intervals (CI), median and interquartile range or numbers and percentages as appropriate. We used multivariable adjusted logistic regression to test whether U-IgM excretion (detectable compared with non-detectable urine IgM) and increasing urinary albumin excretion (U-ACR > 2.9, U-ACR 1.5–2.9 and U-ACR 0.1–1.4 mg/mmol compared with non-detectable urine albumin) were related to vascular parameters. In a multivariable logistic regression model, we adjusted for predefined variables; age, sex, BMI, eGFR, current smoker (risk factors for CV disease) and antihypertensive treatment (hypertension is a risk factor for CV disease and antihypertensive drugs may affect glomerular filtration of albumin, and IgM). Smoking status was known from the web-based questionnaire in 1323/1531 (86.4%) subjects. In the multivariable logistic regression model, subjects who did not answer the questionnaire were treated as non-smokers, adopting a conservative approach. To test if detectable urinary IgM was associated with increasing levels of urinary albumin excretion (non-detectable urine albumin, low normoalbuminuria, high normoalbuminuria, or microalbuminuria), a multivariable logistic regression model was used, adjusted for age and sex. Reported *p*-values are 2-tailed and the level of significance was set at *p* < 0.05. All analyses were performed in the SPSS statistical package (v24; SPSS Inc., Chicago, Ill).

## Results

### Participant baseline characteristics

In this sub-study from MOS, 1531 subjects where included and the age of the study population was mean 39 years (95% CI 39-40y, SD 13y, range 18-69y). Among these, 740 were men (48%) and 791 women (52%). There were 197 (12.9%) current smokers and 329 (21.5%) previous smokers in the whole cohort. The mean body mass index (BMI) was 25.8 kg/m^2^ (95% CI: 25.6–26.1; SD 4.7). Thirty-eight (2.4%) subjects had diabetes mellitus, 70 (4.6%) were on anti-hypertensive treatment and 31 (2.0%) had lipid-lowering treatment. Twelve (0.8%) subjects had history of acute myocardial infarction (AMI) and 12 (0.8%) had history of stroke at baseline. In 838 (54.7%) of subjects, urinary albumin could not be detected, 624 (40.8%) had U-ACR levels between 0.1 and 1.4 mg/mmol, 40 (2.6%) between 1.5 and 2.9, and 27 (1.8%) had U-ACR levels ≥3.0. In 76 of 1339 (5.7%) subjects, IgM was detected in urine and the median urinary IgM/creatinine ratio was 1.56 μg/mol, with the 25th–75th percentile ranging from 0.90–2.57 μg/mol). Urinary IgM was not detected in 1263 of 1339 (94.3%) subjects. Baseline characteristics based on detectable IgM or not, as well as non-detectable urine albumin, low- or high normoalbuminuria and microalbuminuria, are shown in Table 1.

Based on means and 95% CI, medians and IQR, numbers and percentages, there were no apparent associations between self-reported diabetes, previous cardiovascular events or eGFR and normoalbuminuria, microalbuminuria or detectable urine IgM in the cohort (Table 1). However, age and the proportion of subjects with self-reported smoking, hypertension and antihypertensive treatment, and the mean systolic and diastolic blood pressures, were higher among subjects with microalbuminuria compared to subjects with non-detectable urine albumin (Table 1). The proportion of subjects with antihypertensive treatment was also slightly higher among subjects with IgM-uria and those with high grade normoalbuminuria compared to subjects without detectable urine IgM and albumin, respectively.

Detectable urinary IgM was associated with increasing levels of urinary albumin excretion, OR 1.4 (95% confidence interval 1.1–1.8, *p* = 0.002).

### Vascular markers and endothelial function in relation to urinary albumin- and IgM-excretion

In a multivariable logistic regression model where adjustment was made for predefined variables: age, sex, BMI, eGFR and current smoker, detectable urine IgM was significantly associated with higher systolic blood pressure and lower ABI in both models. Further adjustment for systolic blood pressure did not change the results (data not shown). Microalbuminuria and high normoalbuminuria were in the same models associated with systolic and diastolic blood pressure of the brachial artery and systolic blood pressure of the aorta, as well as the aortic c-f PWV and the number of carotid intima plaques. Neither urinary IgM excretion or microalbuminuria, nor high or low normoalbuminuria was associated with the RHI (Table 2).

**Table 2:** Odds ratios with 95% confidence intervals (95%CI) of having detectable urinary IgM excretion compared to not detectable, and the odds of microalbuminuria, high or low normoalbuminuria compared to not detectable urinary albumin excretion. Analyses were adjusted for age, sex, BMI, eGFR, current smoking and antihypertensive treatment. High normoalbuminuria was defined as ACR 1.5–2.9 mg/mmol and low normoalbuminuria was defined as ACR 0.1–1.4 mg/mmol

	Urinary IgM excretion		Urinary albumin excretion					
	Urinary IgM ≥ 10 ng/L (*n* = 76)		U-ACR ≥ 3.0 mg/mmol (*n* = 27)		U-ACR 1.5–2.9 mg/mmol (*n* = 40)		U-ACR 0.1–1.4 mg/mmol (*n* = 624)	
	OR (95% CI)	p	OR (95% CI)	p	OR (95% CI)	p	OR (95% CI)	p
Systolic BP	1.021 (1.003–1.039)	**0.025**	1.064 (1.033–1.097)	**< 0.001**	1.050 (1.025–1.075)	**< 0.001**	1.026 (1.017–1.036)	**< 0.001**
Diastolic BP	1.029 (1.000–1.060)	0.050	1.095 (1.047–1.145)	**< 0.001**	1.078 (1.038–1.119)	**< 0.001**	1.041 (1.026–1.056)	**< 0.001**
24-h mean SBP	1.023 (0.995–1.051)	0.114	1.077 (1.033–1.122)	**< 0.001**	1.083 (1.044–1.124)	**< 0.001**	1.026 (1.012–1.041)	**< 0.001**
24-h mean DBP	1.025 (0.986–1.064)	0.210	1.097 (1.035–1.163)	**0.002**	1.090 (1.038–1.145)	**0.001**	1.026 (1.008–1.046)	**0.006**
24-h mean central systolic BP	1.005 (0.981–1.029)	0.703	1.063 (1.019–1.108)	**0.005**	1.062 (1.022–1.104)	**0.002**	1.006 (0.996–1.016)	0.277
24-h mean aortic c-f PWV	0.968 (0.744–1.259)	0.810	1.887 (1.114–3.197)	**0.018**	2.120 (1.352–3.323)	**0.001**	1.119 (0.983–1.274)	0.088
Daytime aortic c-f PWV	0.954 (0.745–1.221)	0.706	1.860 (1.080–3.205)	**0.025**	2.066 (1.309–3.260)	**0.002**	1.104 (0.975–1.251	0.120
Nighttime aortic c-f PWV	1.096 (0.871–1.379)	0.434	1.838 (1.131–2.986)	**0.014**	2.010 (1.320–3.063)	**0.001**	1.044 (0.963–1.131)	0.293
RHI	0.822 (0.493–1.371)	0.453	1.582 (0.653–3.833)	0.310	1.626 (0.853–3.102)	0.140	1.242 (0.982–1.572)	0.071
ABI dx	0.026 (0.002–0.381)	**0.008**	0.038 (0.000–3.372)	0.153	3.936 (0.128–121.26)	0.433	1.296 (0.382–4.4059	0.677
ABI sin	0.040 (0.003–0.496)	**0.012**	0.142 (0.003–7.674)	0.338	3.571 (0.094–135.00)	0.492	2.312 (0.665–8.043)	0.188
Number of carotid plaques dx	1.040 (0.669–1.617	0.863	1.608 (0.909–2.843)	0.102	1.812 (1.086–3.023)	**0.023**	1.001 (0.811–1.235)	0.995
Number of carotid plaques sin	1.065 (0.696–1.631)	0.770	1.804 (1.090–2.986)	**0.022**	1.391 (0.846–2.287)	0.194	1.057 (0.871–1.283)	0.573

*OR* odds ratio, *CI* confidence interval, *BP* blood pressure, *SBP* systolic blood pressure, *DBP* diastolic blood pressure, *RHI* reactive hyperemia index, *ABI* ankle-brachial-index, *ACR* albumin creatinine ratio, *BMI* body mass index, *eGFR* estimated glomerular filtration rate, *c-f PWV* carotid-femoral pulse wave velocity

## Discussion

The main finding of the present observational study involving young to middle-aged and mostly healthy individuals was that detectable urinary IgM (U-IgM) was associated with lower ankle brachial index (ABI). There was also a weak association between U-IgM and higher systolic blood pressure, however, this association was not observed between U-IgM and 24-h mean systolic blood pressure. Other markers of hypertension, arterial stiffness and endothelial dysfunction, did not associate with U-IgM in the present study. On the other hand, high-grade normoalbuminuria (ACR 1.5–2.9) and microalbuminuria (ACR ≥ 3.0 mmol/mg) were associated with clinical markers of hypertension, arterial stiffness and carotid atherosclerosis, but not RHI (marker of endothelial dysfunction) nor ABI (marker of peripheral atherosclerosis). Low-grade normoalbuminuria (ACR 0.1–1.4 mg/mmol) was associated with higher systolic and diastolic blood pressure. Based on these findings it could be speculated that microalbuminuria and a high normal range of albuminuria of > 1.5 mg/mmol reflects both *arteriosclerosis* (vascular function), i.e. morphological characteristics of the tunica media of large elastic arteries involving decreased elastin and higher collagen content [3], and subclinical *atherosclerosis* (vascular structure), which is associated with narrowing of the arterial lumen secondary to accumulating cells and lipids in the tunica intima [37]. Speculatively, U-IgM may therefore more specifically reflect subclinical peripheral atherosclerosis than arteriosclerosis. Nevertheless, we also found that detectable U-IgM was associated with increasing levels of urinary albumin excretion, suggesting that the pathogenesis underlying increased U-IgM and urinary albumin excretion are partly overlapping.

These results strengthen previous findings suggesting different pathogenesis leading to increased urinary albumin and IgM excretion, respectively. High levels of U-IgM excretion, independently of low grade albuminuria, was associated with increased cardiovascular events and mortality in patients with type 1 and 2 diabetes, but also in patients with acute chest pain [9, 10, 16]. Furthermore, low grade albuminuria to a greater extent reflected systemic inflammation [38], and the strongest predictive value predicting future cardiovascular risk was found when combining low grade albuminuria and U-IgM [9, 10, 38].

With reference to the two-pore theory of glomerular permeability, only a very small amount of albumin (radius 35.5 Å) passes the glomerular filtration barrier (GFB; 1 of 10,000 albumin molecules). Larger proteins, including IgM (radius 120 Å), pass the GFB at much lower rates, and only through large defects (shunts), indicative of severe injury to the GFB [18–20].

In the present study, 6% of the study participants had detectable concentrations of U-IgM, which could suggest that these individuals are afflicted by some degree of structural injury to the GFB. These findings were unrelated to age, sex, eGFR, previous CV events, diabetes and drugs to treat hypertension or hyperlipidemia. However, the presence of urinary IgM was associated with a higher systolic blood pressure and a lower ABI. The latter is a well-known marker of peripheral artery disease and associated with generalized atherosclerosis, CV events and mortality [6–8]. Therefore, it is plausible that the observed relation between urinary IgM and ABI is related to subclinical atherosclerosis leading to elevated glomerular vascular resistance, glomerular ischemia, and remodeling of the GFB with an increased number of non-selective glomerular shunt-pathways [19, 39–41].

Furthermore, 2% of participants had microalbuminuria, 3% had high-grade normoalbuminuria and 41% had low-grade normoalbuminuria. Microalbuminuria is associated with systemic inflammation, endothelial dysfunction. It is a well-known risk marker for atherosclerosis, artery stiffness, cardiovascular morbidity and mortality in subjects with diabetes and in populations with increased cardiovascular risk [12, 13, 42, 43]. Later it was shown that these findings also apply to subjects without hypertension or diabetes in middle-aged and elderly individuals. Here we show similar findings in a younger population of mostly healthy individuals. Our findings are in line with several studies which suggest that a U-ACR cutoff below 3 mg/mmol should be used when considered as a risk marker of cardiovascular risk in non-diabetic subjects [11, 13, 15, 44]. We did, however, not find an association between low grade albuminuria and RHI measured in the finger, a marker of endothelial dysfunction, which has been found in other studies of subjects with type 2 diabetes [45]. This could be related to the fact that our study population is younger and mostly healthy, or that the present study was underpowered to detect these differences.

The pathophysiological mechanism linked to increased albumin excretion are multifactorial and mediated by increased renal vascular resistance secondary to atherosclerosis, arteriosclerosis and hypertension, chronic inflammation, increased RAAS activity and dysfunction of other hormonal systems [46–49]. Hence, increased urinary albumin excretion as a marker of permanent glomerular/endothelial dysfunction and increased cardiovascular risk has some limitations because it varies depending on systemic inflammatory and hormonal status [46, 47, 49]. This study extends previous investigations by showing associations between the level of urinary albumin excretion and hypertension as well as markers of arterial stiffness and subclinical atherosclerosis in young and mostly healthy individuals. Therefore, microalbuminuria and high normoalbuminuria could be predictive of subsequent cardiovascular morbidity also in younger mostly healthy individuals, to be further explored in our cohort. The different molecular sizes of albumin (molecular radius 35.5 Å) and IgM (molecular radius 120 Å) explains differences in excretion since elimination of IgM through the GFB requires a more extensive barrier damage compared to albumin. This may explain differences in the effect on the evaluated atherosclerotic vascular parameters in this study. The presence of IgM in the urine could demonstrate another or more severe damage of the GFB. Therefore, further evaluation of this phenomenon may lead to a better understanding of the significance of the urinary filtration quality. However, quantification of proteinuria is complicated due to reabsorption in the tubular part. Overloading of the reabsorption capacity leads to measurable proteinuria. Reabsorption capacity of albumin and IgM may differ and jeopardize the interpretation.

There are some strengths and limitations of our study to be acknowledged. The community-based study population of the present study has been well characterized which is an important strength. In this study population of younger, mostly healthy individuals there was only a small percentage of subjects with low grade albuminuria (2%) and detectable U-IgM (6%). This limits our study by precluding more extensive multivariable analyses, why we decided to only include age, sex, BMI, eGFR, smoking and anti-hypertensive treatment in the multivariable analyses. To various degree the subjects had not underwent all technical examinations used in MOS, and 15% had not filled the questionnaires which is a limitation of the study. Also, the detection limit of our U-IgM assay, in combination with the level of concentration of IgM in the primary urine, influences which subject for whom we can detect IgM in the urine. This must be considered a weakness. Another weakness of the study is that information about pharmacological treatment and medical history was gained from a self-administered questionnaire. Also, U-IgM may not only be related to the glomerular permeability, but also to the degree of tubular reabsorption/excretion and discharge from the bladder, urethra etc.

## Conclusions

When measured in younger, mostly healthy individuals, increased urinary IgM excretion and low-grade albuminuria are associated with vascular parameters reflecting vascular ageing and increased risk of cardiovascular morbidity and mortality. This study suggests that increased urinary IgM may be a more specific marker of atherosclerosis (associates with ABI), whereas increased albumin excretion could be mediated or reflected by a wide variety of cardiovascular abnormalities. By measuring both urinary albumin and IgM excretion (in combination with other known risk factors), the prognostic ability to estimate the overall cardiovascular risk could be increased and also reflect different targets of the examined vascular parameters. This needs to be addressed in future prospective investigations.

## Supplementary information

**Additional file 1: Supplementary Table 1.** The proportion of subjects that have undergone the various examinations performed in the present study

## Data Availability

All data generated or analyzed during this study are included in this published article. Corresponding author (PS) can be contacted for more information.

## Abbreviations

CI: Confidence interval
AMI: Acute myocardial infarction
IQR: Interquartile range
RAAS: Renin-angiotensin-aldosterone system
eGFR: Estimated glomerular filtration rate
ABI: Ankle-brachial-index
ACR: Albumin creatinine ratio
BMI: Body mass index
c-f PWV: Carotid-femoral pulse wave velocity
RHI: Reactive hyperemia index
MOS: Malmö offspring study
U: Urine
GFB: Glomerular filtration barrier
CV: Cardiovascular
