# Supplementary material for: Patterns of urinary albumin and IgM associate with markers of vascular ageing in young to middle-aged individuals in the Malmö offspring study
Source: BMC Cardiovasc Disord. 2020 Aug 5;20:358. doi: 10.1186/s12872-020-01638-3 (PMC7409481; doi:10.1186/s12872-020-01638-3)
Supplement: Supplementary file 1 — Additional file 1: Supplementary Table 1. The proportion of subjects that have undergone the various examinations performed in the present study [file 12872_2020_1638_MOESM1_ESM.docx]

Supplementary table 1. The proportion of subjects that have undergone the various examinations performed in the present study

| Variable | N (% of 1531) |
| --- | --- |
| weight | 1531 (100) |
| height | 1531 (100) |
| BMI | 1531 (100) |
| Waist circumference | 1531 (100) |
| Blood pressure | 1518 (99.1) |
| Questionnaire | 1323 (86.4) |
| ABI | 1522 (99.4) |
| c-f PWV | 546 (35.6) |
| arteria carotis morphology | 659 (43.0) |
| 24-h blood pressure | 834 (54.5) |
| RHI | 1009 (65.9) |
| p-creatinine | 1528 (99.9) |
| eGFR | 1513 (98.8) |
| blood lipids | 1529 (99.9) |
| HbA_1c_ | 471 (30.8) |
| u-ACR | 1530 (99.9) |
| u-IgM | 1339 (87.5) |

BMI: body mass index; ABI: ankle-brachial-index; c-f PWV: carotid-femoral pulse wave velocity; RHI: reactive hyperemia index; ACR: albumin creatinine ratio.
